# microRNAs 424 and 503 are mediators of the anti-proliferative and anti-invasive action of the thyroid hormone receptor beta

**DOI:** 10.18632/oncotarget.1577

**Published:** 2014-04-02

**Authors:** Lidia Ruiz-Llorente, Soraya Ardila-González, Luisa F Fanjul, Olaia Martínez-Iglesias, Ana Aranda

**Affiliations:** Instituto de Investigaciones Biomédicas, Consejo Superior de Investigaciones Científicas and Universidad Autónoma de Madrid, Arturo Duperier 4, 28029 Madrid, Spain

**Keywords:** thyroid hormone receptor, microRNA-424, microRNA-503, proliferation, invasion, hepatocarcinoma and breast cancer cells

## Abstract

The thyroid hormone receptors (TRs) mediate tumor suppressive effects in hepatocarcinoma and breast cancer cells. Here we show that incubation of hepatocarcinoma SK-hep1 cells expressing TRb with the thyroid hormone T3 induces transcription of the polycistronic message coding for microRNAs 424 and 503. TRb binds to the promoter region of these miRNAs and T3 induces an exchange of corepressors and coactivators inducing histone acetylation and transcriptional stimulation. We have validated cell cycle components as targets of these miRNAs. Overexpression of miR-424 mimicked the repressive effect of T3 on cell proliferation, growth in suspension, migration and invasion. Knockdown of miR-424 or miR-503 reduced the inhibitory effect of the hormone. T3 increased miR-424 and miR-503 in breast cancer cells expressing TRb, and this induction is also involved in the anti-invasive effects of the hormone. Furthermore, miR-424 or miR-503 depletion enhanced extravasation to the lungs of hepatocarcinoma cells injected in the tail vein of mice. The levels of these miRNAs were reduced in xenograft tumors formed in hypothyroid nude mice that are more invasive. Therefore, miR-424 or miR-503 mediate anti-proliferative and anti-invasive actions of TRb both in cultured cells and *in vivo*.

## INTRODUCTION

The actions of the thyroid hormone triiodothyronine (T3) are mediated by binding to nuclear receptors (TR alpha and beta), which act as ligand-dependent transcription factors by association to thyroid hormone response elements (TREs) in target genes [1]. TRs play an important role in cell proliferation and cancer [2, 3]. In hepatocarcinoma cells expressing TRb, T3 reduces proliferation [4], the responses to the *ras* oncogene [5], and the expression of pituitary tumor-transforming 1, a critical mitotic checkpoint protein [6]. Thyroid hormone treatment induces regression of carcinogen-induced hepatic nodules, reducing the incidence of hepatocarcinoma and lung metastasis in rodents [7, 8]. Furthermore, decreased TR levels and somatic mutations in TR genes have been found in more than 70% of human hepatocarcinomas, and most of these mutants act as dominant-negative inhibitors of TR activity [9-12]. Inactivation of TRb by promoter methylation, mutations, altered expression and anomalous subcellular localization of TRs has also been described in breast tumors [13-15]. These observations suggests that native TRs could act as tumor suppressors, and indeed expression of TRb in hepatocarcinoma and breast cancer cells retards tumor growth and strongly reduces invasion, extravasation and metastasis formation in nude mice [16, 17]

MicroRNAs (miRNAs) are single-stranded RNA molecules of 20-23 nucleotides length that post-transcriptionally control gene expression [18]. miRNAs bind to 3' untranslated regions (3' UTRs) of mRNA transcripts and promote mRNA degradation or translational inhibition [19-22]. Many miRNAs have oncogenic or tumor suppressive actions [23-25]. Among them, the miR-16 family regulates cell proliferation [26-28] and miR-503, a miR-16 family member, might be a master regulator of the cell cycle [29]. miR-503 is an intragenic miRNA clustered with miR-424, other miR-16 family member, and both are produced as a polycistronic message [30]. Various targets of these miRNAs regulate cell division, the cell cycle, mitosis or angiogenesis [31-37]. In addition, miR-424 and miR-503 are involved in cancer cell migration and invasion [38, 39], and are reduced in human hepatocarcinoma tumors [40].

In this work we show that miR-424 and miR-503 are transcriptionally induced by T3 in hepatocarcinoma and breast cancer cells expressing TRb, and demonstrate that this induction appears to play an important role in the anti-proliferative and anti-invasive actions of the hormone both in cultured cells and *in vivo*.

## RESULTS

### T3 induces transcriptional activation of miR-424 and miR-503

**Figure 1:**
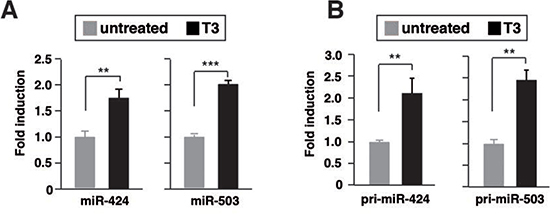

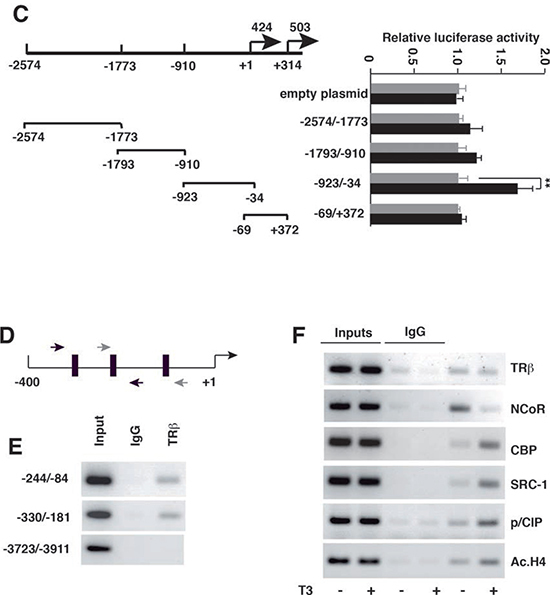
T3 induces transcription of miR424 and miR503 in hepatocarcinoma cells **(A)** miR-424 and -503 levels after 48 h of treatment with 5nM T3 in TRb-expressing SK-hep1 cells. Fold change of these microRNAs are expressed relative to control untreated cells. **(B)** pri-miRNA levels measured under the same conditions. **(C)** Schematic diagram of the 5'-flanking region of miRNA424/503, and the fragments used in transient transfection studies. Cells were transfected with the empty luciferase plasmid or with the plasmids containing the indicated regions of the miRNA-424/-503 promoter. Luciferase activity was determined in control cells and in cells treated with T3 for 48 h. **(D)** Schematic representation of the miRNA-424/-503 proximal promoter depicting the position of the putative receptor binding sites (black boxes) and the primers used in chromatin immunoprecipitation (ChIP) assays (arrows). **(E)** ChIP assays with IgG and TRb antibodies and the indicated fragments of the miRNA-424/-503 promoter. **(F)** Binding of TRB, the corepressor NCoR, the coactivators CBP, SRC-1 and p/CIP and acetylated histone H4 to the miRNA-424/-503 promoter region −244/−84, determined by ChIP assays after 1 h of incubation with T3.

In miRNA microarrays we found that miR-424 and miR-503 levels were higher (2.32- and 2.99-fold, respectively) in T3-treated SK-hep1 cells expressing TRb (SK- TRb) than in untreated cells. The expression levels of both miRNAs measured by quantitative RT-PCR were increased upon T3 treatment, validating the microarrays results (Figure 1A). This increase was not found in parental cells that do not express TRb (Suppl. Figure 1), indicating that the effect of T3 requires binding to the receptor. Expression of miR-424 and miR-503 was, however, undetectable in parental and TRb-expressing HepG2 cells both in the absence and presence of T3 (Suppl. Figure 2A,B). In addition, T3 did not increase the levels of these miRNAs in non-transformed THLE-2 hepatocytes. However, the hormone induced miRNA expression in HH4 hepatocytes, and this induction was stronger after transduction with a retroviral vector encoding TRb (HH4-TRb cells) (Suppl. Figure 2B). These cells express the receptor at similar levels as those found in SK-TRb or HepG2-TRb cells (Suppl. Figure 2B). Therefore, induction of miR-424 and miR-503 by T3 is not restricted to hepatocarcinoma cells.

T3 increased the levels of pri-miRNA-424 and pri-miRNA-503 in SK- TRb cells (Figure 1B), suggesting that the hormone induces transcription of the miRNAs precursor. To analyze the presence DNA elements that could mediate T3-dependent transcription, we constructed luciferase reporter plasmids containing different fragments of the miRNAs 5'-flanking region (Figure 1C). Activity of the –923/-34 region with respect to the transcription start site of miR-424 was increased by T3. However, no change was observed when cells were transfected with the luciferase plasmid alone or with other fragments, indicating that this region contains the elements responsible for stimulation of miR-424/503 by T3.

ChIP assays were carried out with the region containing the putative response elements (Figure 1D). TRb bound constitutively to two overlapping fragments of the proximal promoter region, whereas no receptor recruitment to an irrelevant upstream region (-3723-/3911) was observed (Figure 1E). Furthermore, T3 caused dissociation of the corepressor NCoR and recruitment of p160 coactivators and CBP to the promoter. Consequently, enrichment on acetylated H4 histone was also detected (Figure 1F).

### T3 down-regulates miR-424 and miR-505 targets

**Figure 2:**
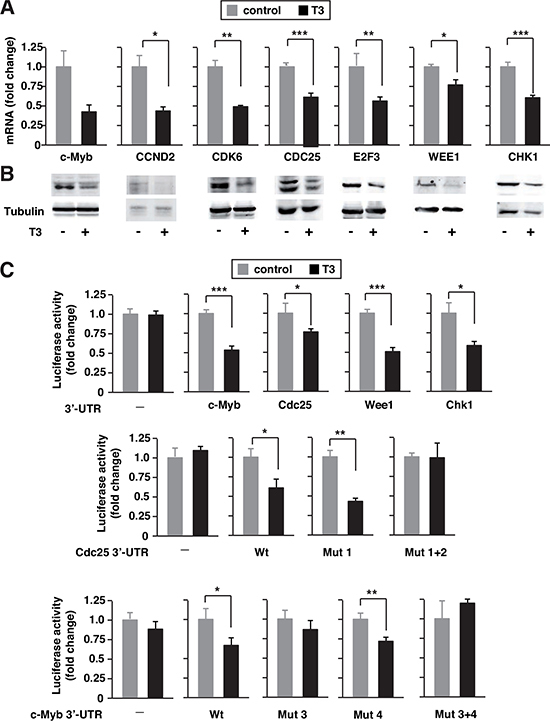
T3 down-regulates expression of miRNA424/503 targets **(A)** mRNA levels for the indicated putative targets of miRNAs 424 and 503 were measured by quantitative PCR in SK-TRb cells treated without and with T3 for 48 h. Fold change of these mRNAs, normalized to RPL19 mRNA, are expressed relative to the values obtained in control untreated cells. **(B)** Western blot analysis of the same targets. Tubulin was used as a loading control. **(C)** Luciferase reporters fused to the c-Myb, Cdc25, Wee1 and Chek1 3,-UTRs, as well as an empty plasmid, were transfected into SK-TRb cells. A mutant construct of the proximal binding site in the Cdc25 3'-UTR is designated Mut1, the mutation in the distal binding site Mut2, and the mutation of both binding sites Mut 1+2. Mut3 indicates mutation of the distal binding site in the c-Myb-3'-UTR, Mut4 of the proximal site and Mut 3+4 of both sites. Luciferase activity was measured after 36h incubation in the presence and absence of T3.

miR-424 and miR-503 are predicted to target many mRNAs (microRNA and TargetScan), some of them already validated [31, 37, 41]. In SK-TRb cells, T3 down-regulated mRNA levels of Ccnd2, Cdk6, Cdc25, E2f3, c-Myb, Wee1 and Chk1 involved in cell proliferation and cancer (Figure 2A). Furthermore, the levels of these proteins, assessed by western blot, were reduced in T3-treated cells (Figure 2B). This is compatible with the hormone-dependent induction of miRNAs 424 and 503.

The 3**′-**UTRs of the tested mRNAs have sequences that could anneal to the seed sequences of miR-424 and 503 (Suppl. Figure 3). To validate the predicted miRNA/mRNA interactions, we used luciferase vectors containing the 3**'-**UTR of c-Myb, Cdc25A, Wee1 and Chk1. T3 did not affect the activity of the plasmid without UTR sequences, but significantly inhibited the activity of the UTR-containing constructs (Figure 2C). Therefore, T3 appears to represses target mRNA stability as a consequence of the increased levels of miR-424/503.

Previously described mutants of the putative miR-424/503 binding sites in the Cdc25 and c-Myb UTRs were used to analyze induction by T3. Cdc25 3'-UTR contains two binding sites for these miRNAs [41]. Mutation of the first site did not affect inhibition by T3. However, mutation of both sequences abrogated the repression by the hormone (Figure 2C). This shows that the reported binding sites for miR-424/503 are relevant for the effect of T3. The c-Myb UTR also contains two putative sites for these miRNAs. Mutation of the distal site alone or in combination with the proximal site abolished responsiveness to T3 (Figure 2C), in agreement with the finding that this binding site mediates the effect of miR-424 [42].

**Figure 3:**
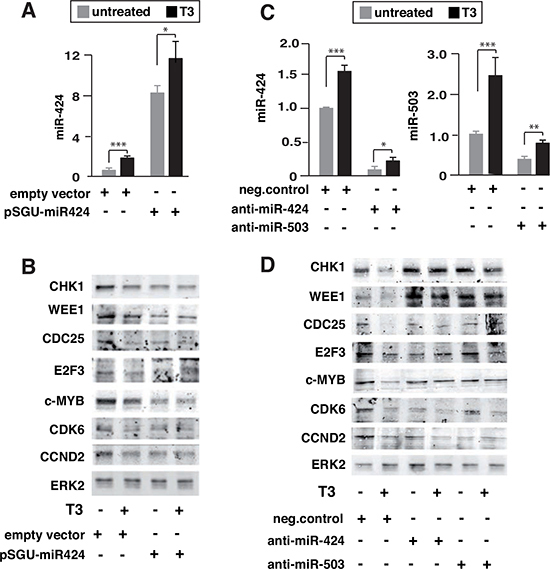
Influence of miR-424 expression and miR-424 and miR-503 depletion on the effect of T3 on target proteins **(A)** miR-424 levels in untreated and T3-treated cells transfected 36 h before with an expression vector for miR-424 (pSGU-miR424) or the corresponding empty vector. **(B)** The levels of CHK1, WEE1, CDC25, E2F3, c-MYB, CDK6 and CCND2 were measured by western blot. ERK2 levels were used as a loading control. **(C)** SK-TRb cells were transfected with anti-miRNAs for miR-424 or miR-503, or with a negative control, and the levels of the corresponding miRNAs were determined in untreated and T3-treated cells 36 h after transfection. **(D)** Levels of the indicated proteins were analyzed by western blot under the same conditions.

To further test the functional relevance of miR424 and miR-503 induction by T3, we conducted experiments of gain and loss of function of the miRNAs and evaluated the expression of the different targets by western blotting. Over-expression of miR-424 (Figure 3A) mimicked the effect of T3, decreasing their levels (Figure 3B). Furthermore, the reduction caused by T3 and miR-424 was not additive, indicating a common mechanism of action. On the other hand, transfection with miRNA inhibitors, which caused a strong reduction of miR-424 or miR-503 levels (Figure 3C), reversed the inhibitory effect of T3 on CHK1, WEE1, CDC25, c-MYB or E2EF, while CDK6 or CCND2 levels remained low (Figure 3D).

### miR424/503 depletion antagonizes the anti-proliferative and anti-invasive actions of T3

**Figure 4:**
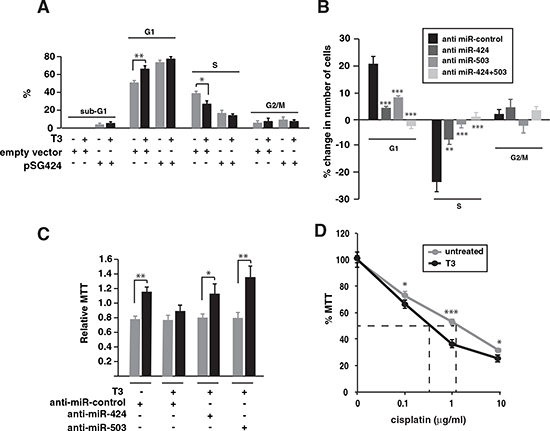
Inhibition of miR-424 and -503 reverses the effect of T3 on SK-TRb cell proliferation **(A)** Cell cycle analysis in cells transfected with pSGU-miR424 or an empty vector. DNA contents were measured after 48 h of treatment with and without T3. The results are shown as the % of cells in sub-G1, G1, S and G2/M phases. **(B)** Changes in the cell cycle after transfection with the negative control RNA, anti miR-424, anti miR-503 or both. The percentage of change in T3 versus untreated cells for each condition was plotted. **(C)** Cells were transfected with the indicated anti-miRNAs and after 24h were detached from the plates and grown under rocking conditions during an additional 24 h period in the presence or absence of T3. Cells were then plated and MTT was assayed. Results were expressed relative to MTT values obtained in cells inoculated in parallel without agitation. **(D)** MTT values in cells treated with or without T3 for 72 h and with the indicated concentrations of cisplatin for an additional 48 h period.

Since miR-424 and miR-503 down-regulate proteins with an important role in cell proliferation, we analyzed their effect on the cell cycle of SK-TRb cells. Transfection of miR-424 increased the percentage of cells arrested in G1, causing a concomitant reduction in the number of cells in S-phase and a small increase of sub-G1 cells. Incubation with T3 did not induce cell death, but caused G1 increase and reduction of S-phase, although less marked than that caused by miR-424 overexpression. In addition, the hormone had little effect in cells overexpressing the miRNA (Figure 4A). Depletion of miR-424 or miR-503 strongly inhibited the effects of T3, and combined knockdown of both miRNAs abolished T3-dependent increase in G1 and reduction of S-phase. This indicates the crucial role of miR-424/503 induction for this hormonal effect (Figure 4B).

TRb expression prevents the ability of SK-hep1 cells to grow in the absence of a solid substrate [17]. Therefore, we next examined the effect of miR-424 and miR-503 on growth of SK-TRb cells in suspension under rocking conditions. miR-424 overexpression mimicked the effect of T3 inhibiting growth in suspension, while depletion of either miR-424 or miR-503 reversed T3-dependent inhibition (Figure 4C). Thus, miR-424 and miR-503 induction is also involved in this action of T3 in SK-TRb cells. Transfection of miR-424 also reduced growth in suspension of HepG2-TRb cells that do not express miR-424 or miR-503 (Suppl. Figure 4A). In contrast, in these cells T3 did not reduce growth and, as expected, the miRNA inhibitors were ineffective (Suppl. Figure 4B).

Increased expression of Wee1 and Chk1 secondary to reduced miR-424 and other family members has been associated with resistance to cisplatin [36]. In agreement with this finding, T3 caused a moderate but statistically significant reduction of SK-TRb cell survival in response to the drug, with the EC50 decreasing from 1.6 µg/ml to 0.8 µg/ml (Figure 4D).

**Figure 5:**
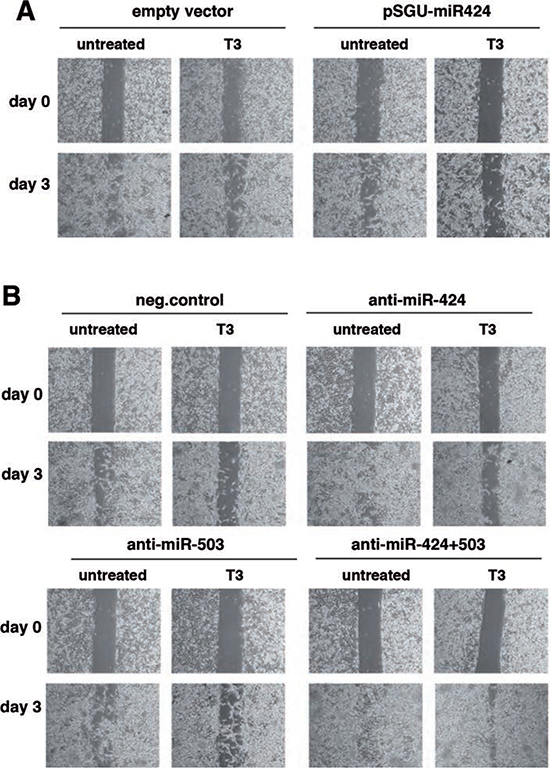
miR-424 and -503 are involved in the inhibitory effect of T3 on hepatocarcinoma cell migration **(A)** Cells transfected with pSGU-miR424 or the empty vector were treated with mitomycin C to block proliferation and wound healing was followed for 3 days in the presence and absence of T3. Photographs were taken at time 0 and at day 3. Representative images are shown. **(B)** Similar experiment performed in cells transfected with a control anti-miRNA, or anti-miR-424 and anti-miR-503 alone or in combination.

We next studied whether T3-induced miRNA increase could also affect cellular migration. T3 treatment or transfection with pSGU-miR424 retarded SK-TRb cell migration in wound assays (Figure 5A). Conversely, miR-424 or miR-503 depletion increased wound closure and, when used in combination, reversed the inhibitory effect of T3 (Figure 5B). Therefore, induction of miRNAs 424 and 503 appears to mediate the inhibitory effect of T3 on hepatocarcinoma cell migration.

**Figure 6:**
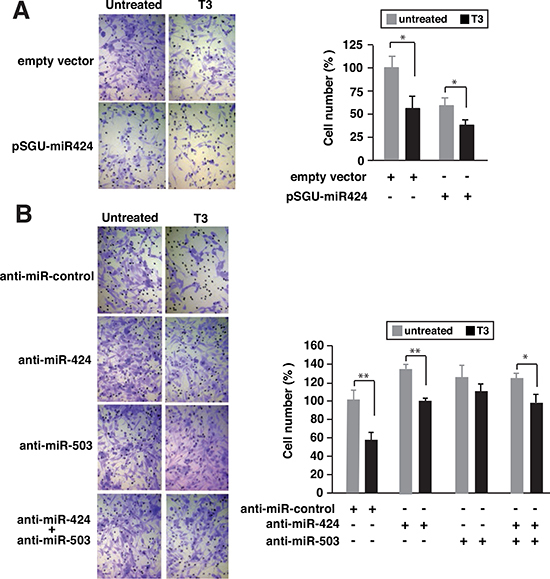
Depletion of miR-424 and miR-503 reduces the inhibitory effect of T3 on cellular invasion **(A)** SK-TRb cells were transfected with pSGU-miR424 or the empty vector and incubated for 48 h in the presence and absence of T3. Cells were then inoculated into the upper chamber of Transwell plates containing matrigel and treated with or without T3. Sixteen hours later cells that passed through the filter were stained and scored. Representative images are shown at the left and quantifications at the right panel **(B)** Similar experiment in cells transfected with a negative control anti-miRNA, anti-miR-424, anti-miR-503 or both.

Our next objective was to investigate the possible role of these miRNAs on the reduced invasive capacity shown by T3-treated SK-TRb cells [17]. Figure 6A shows that over-expression of miR-424 again mimicked the effect of T3, reducing cellular invasion through matrigel. Furthermore, depletion of miR-424, miR-503, or both, increased cellular invasion and reduced noticeably the inhibitory effect of T3. Therefore, induction of these miRNAs by T3 also appears to play a role on the anti-invasive actions of the hormone (Figure 6B).

### miR424/503 depletion increases extravasation of hepatocarcinoma cells

**Figure 7:**
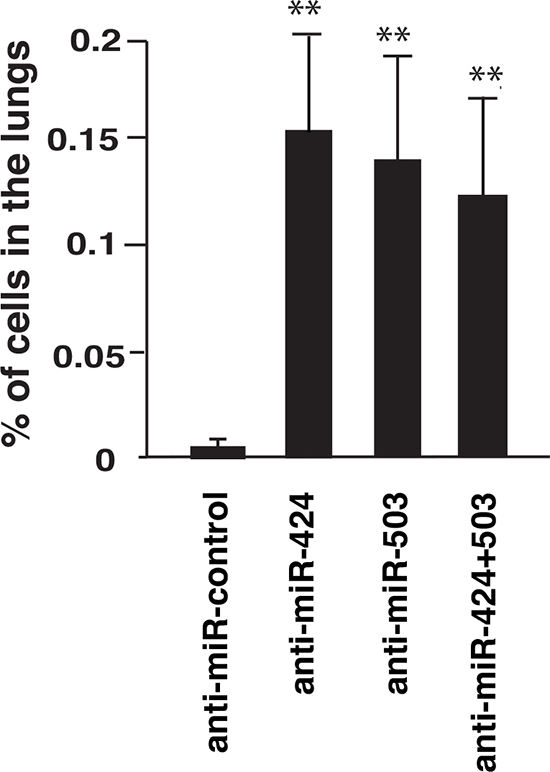
Depletion of miRNAs 424 and 503 enhances extravasation *in vivo* SK-TRb cells transfected with a negative control or with the indicated anti-miRNAs were injected into the tail vein of nude mice. The presence of human *Alu* sequences in the lungs was determined by quantitative PCR at 9 h after injection. Data are expressed as the percentage of the inoculated DNA with respect to the total DNA injected.

TRb can limit cancer cell extravasation *in vivo* [17]. Therefore we next determined if miR-424 and miR-503 could also regulate this process. To analyze this, SK- TRb cells transfected with a negative control of with anti-miRs were injected into the tail of nude mice. As illustrated in Figure 7, miRNA depletion increased very significantly the amount of cells present in the lungs of the mice. Therefore, the induction of miRNAs 424 and 503 by endogenous thyroid hormones could inhibit cell extravasation *in vivo*.

### T3 increases miR424 and 503 levels in breast cancer cells

**Figure 8:**
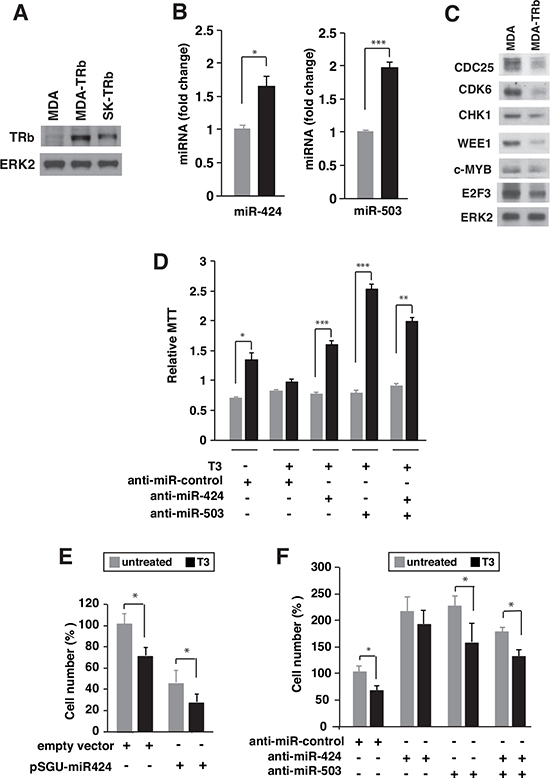
T3 induces expression of miR424 and miR503 in breast cancer cells **(A)** Western blot analysis of TRb in parental MDA-MB-468 (MDA), TRb-expressing MDA (MDA-TRb) cells and SK-TRb cells. ERK2 was used as a loading control. **(B)** miR-424 and -503 levels in MDA-TRb cells after 48 h of treatment with 5nM T3. Fold change of these microRNAs are expressed relative to control untreated cells. **(C)** Western blot analysis of the putative miR424/503 targets CDC25, CDK6, CHK1, WEE1, c-MYB, and E2F3 in untreated and T3-treated MDA-TRb cells. ERK2 was used as a loading control. **(D)** Growth in suspension under rocking conditions was analyzed in MDA-TRb cells previously transfected with the indicated anti-miRNAs. MTT was assayed after 24 h growth in the presence and absence of T3. Results were expressed relative to MTT values obtained in cells inoculated in parallel without agitation. **(E)** Invasion through matrigel was measured in MDA-TRb cells previously transfected with pSGU-miR424 or the empty vector and incubated for 48 h with and without T3. Invasion was then performed for 16 h in the presence and absence of the hormone and the cells that passed through the filter were counted **(F)** Similar experiment in cells transfected with a negative control anti-miRNA, anti-miR-424, anti-miR-503 or both. The results are expressed relative to the number of cells obtained in the control untreated cells.

To analyze if induction of the miRNAs by T3 could also occur in other types of tumor cells, we measured the levels of miRNAs 424 and 503 in TRb-expressing MDA-MB-468 breast cancer cells (MDA-TRb) cells (Figure 8A). T3 increased the levels of both miRNAs (Figure 8B), and in agreement with this induction reduced the levels of their putative target proteins (Figure 8C). The functional role of miR-424 and miR-503 induction by T3 in MDA-TRb cells was also examined. Growth in suspension was abolished by T3 and was restored after depletion of miR-424, miR-503, or both (Figure 8D). The hormone also decreased MDA-TRb cells invasion through matrigel and expression of miR-424 had a similar effect (Figure 8E). In addition, miR-424 and/or miR-503 depletion increased cellular invasion and reduced the inhibition by T3 (Figure 8F). Therefore, induction of miRNAs 424 and 503 by T3 is also functionally relevant in breast cancer cells.

### Hypothyroidism reduces miR-424 and miR-503 levels in xenografts

**Figure 9:**
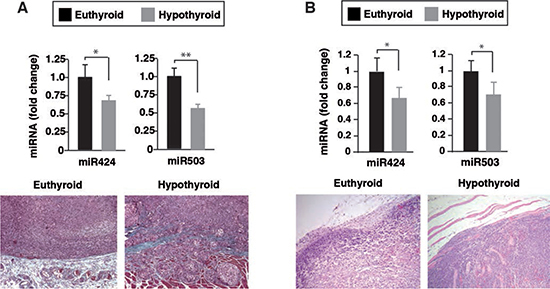
Reduced miR-424 and miR-503 expression in tumor xenografts developed in hypothyroid mice Euthyroid and hypothyroid nude mice were injected with SK-TRb **(A)** and MDA-TRb cells **(B)**, and the levels of miR-424 and miR-503 were determined 8 weeks later in the tumors. Representative Masson's trichrome staining of tumors formed in euthyroid and hypothyroid mice are illustrated in the bottom panels.

We have previously shown that when SK-TRb or MDA-TRb cells were inoculated into hypothyroid nude mice, tumors were more invasive and metastatic growth was enhanced [16]. To analyze whether thyroidal status could also alter miRNA expression in the tumors, we next compared miR-424 and miR-503 levels in xenografts formed by inoculation of SK-TRb and MDA-TRb cells into euthyroid and hypothyroid mice. As shown in Figure 9, miR-424 and miR-503 expression was reduced in the tumors developed in hypothyroid mice, correlating with their increased invasive properties. Thus, the thyroid hormones can regulate expression of these miRNAs *in vivo* in hepatocarcinoma and breast cancer cells.

## DISCUSSION

In the present study, we have investigated the function of miR-424 and miR-503 in the response of hepatocarcinoma to T3. The hormone increased the levels of these miRNAs in SK-TRb cells and this induction plays an important role in the anti-tumorigenic and anti-invasive actions mediated by binding of T3 to the receptor. Induction of these miRNAs by T3 was also found in non-transformed hepatocytes and in MDA- TRb breast cancer cells, indicating that the phenomenon is not specific for the hepatocarcinoma cell line.

T3 increased the level of both pri-miRNAs and stimulated the activity of the proximal promoter of miR-424/miR-503 in SK-TRb cells, indicating that the hormone induces transcription of the polycistronic message that encodes both miRNAs. T3-dependent transcriptional stimulation of target genes involves binding of the TR to TREs, inducing the release of corepressors and the recruitment of co-activators that lead to local alteration of chromatin structure [1]. ChIP analysis confirmed that TRb binds to the miR-424/503 promoter, and that T3 releases the corepressor NCoR and recruits p160 coactivators such as SRC-1 or p/CIP with histone acetyltransferase (HAT) activity. p160 coactivators act as primary coactivators interacting with TRs, but they also recruit secondary coactivators such as the HAT CBP/p300 [43]. We observed hormone-dependent recruitment of CBP to the miR-424/503 promoter. Histone acetylation is a critical step in nuclear receptor-mediated hormone signaling and histone acetylation of the miR-424/503 promoter was also induced upon T3 treatment.

miR-424 and 503 play an important role in tumorigenesis. They are down-regulated in several tumors, suggesting that these miRNAs have tumor suppressive activity [37, 39, 42, 44, 45], although miR-424 is upregulated in some tumors [46]. The anti-tumorigenic actions of miR-424 and miR-503 could be related to their effect on the cell cycle. Indeed, elevated levels of both promote cell quiescence and differentiation, which is consistent with the role of this family in targeting multiple genes involved in the G1-S transition [27-29, 33, 41]. Our results show that T3 reduces expression of several cell cycle genes in hepatocarcinoma cells including Ccnd2, Cdk6 and Cdc25 that control entry into and progression through various phases of the cell cycle; the Wee1 kinase, a key regulator during S phase, preventing entry into mitosis until DNA replication has been completed [47]; the checkpoint gene Chk1 [48]; the transcription factor E2f3, a member of the E2f family that regulates expression of genes required for DNA synthesis at the G1/S phase boundary [49]; or the c-Myb proto-oncogene also involved in cell proliferation and cancer [50]. Interestingly, expression of the target proteins of miR-424 and miR-503 was also decreased in T3-treated breast cancer cells.

The increased transcription of miR-424 and miR-503 is relevant for the regulation of most of these proteins by the hormone. On one hand, T3 suppressed stability of target mRNAs in experiments with the 3'-UTRs, which contain binding sites for miR-424/503, and on the other hand, gain- and loss-of-function experiments revealed that these miRNAs mediate T3-dependent changes in the levels of the target proteins. Furthermore, T3 arrested hepatocarcinoma cells in G1 and the knockdown of miR-424 and miR-503 reversed this effect, underscoring the important role of these miRNAs in the inhibition of cell proliferation.

A decrease in the expression of the miR-16 family mediates resistance to cisplatin in cancer cells as a consequence of the increased levels of Wee1 and Chk1 [36]. Our results indicate that T3 significantly repressed Wee1 and Chk1 3**'-**UTR activity and reduced the levels of these proteins. This led us to the hypothesis that elevated expression of miR-424 and 503 in response to T3 could sensitize cancer cells to chemotherapy. In agreement with this idea, we found that hepatocarcinoma cell survival in response to cisplatin was moderately reduced after T3 treatment, suggesting a novel anti-tumorigenic mechanism for this hormone. Down-regulation of Chk1 could also play a role in the anti-tumorigenic effects of TRb, since suppressed miR-424 expression via up-regulation of Chk1 contributes to the progression of cervical cancer [37].

Our results show that miR-424 and 503 affected hepatocarcinoma and breast cancer cell invasion *in vitro*. This is consistent with the previous report that miR-503 was down-regulated in the highly metastatic hepatocarcinoma cell line HCCLM3 when compared with MHCC97-L cells with a lower metastatic potential [39]. The invasive capacity of T3-treated cells was significantly recovered after depletion of miR-424 and 503, indicating that induction of these miRNAs participates in T3-dependent inhibition of SK-TRb and MDA-TRb cell invasion.

The *in vivo* relevance of thyroid hormone regulation of miR-424 and 503 was demonstrated by the finding that tumor xenografts formed by SK-TRb and MDA-TRb cells in hypothyroid mice expressed lower levels of these miRNAs than those developed in animals with normal thyroid function. Interestingly, correlating with the lower levels of miR-424 and 503 the tumors formed in the hypothyroid hosts had a more mesenchymal phenotype and were more invasive [17].

TRb-expressing hepatocarcinoma cells have a reduced capacity of extravasation to the lungs when they are injected into the tail vein of nude mice. Therefore, the receptor appears to have anti-metastatic activity by blocking not only the ability of cancer cells to proliferate and colonize the lung parenchyma, but also by limiting cancer cell extravasation [17]. Here we show that extravasation of hepatocarcinoma cells *in vivo* was strongly enhanced when miRNAs 424 and 503 were depleted. Therefore, induction of these miRNAs could play an important role in the inhibitory action of the receptor. Additionally, miR-424 and miR-503 depletion restored the ability of T3-treated hepatocarcinoma and breast cancer cells to growth in suspension under rocking conditions. The competence to grow under these circumstances should enhance survival of cells in the circulation and therefore increase their ability to metastatize.

Taken together our results show that binding of T3 to the TRb receptor induces transcription of miR-424 and miR-503. This induction is relevant to explain the tumor suppressive actions of this nuclear receptor. The thyroid hormone-dependent increase of miR-424 and miR-503 appears to modulate tumor growth and progression in multiple ways. Besides affecting tumor cell proliferation and increasing sensitivity to DNA damaging agents, elevated levels of miR-424 and miR-503 are required for the inhibitory effect of the hormone on cell migration and invasion. As suggested by our results, the increased miRNA expression could reduce tumor invasion, intravasation, survival of the cancer cells in the bloodstream, and the extravasation and colonization of the metastatized target tissue. There are many predicted targets of miR-424/503 and future studies are necessary to identify the genes responsible for the inhibition of cell migration and invasion by these miRNAs that could affect tumor progression *in vivo*.

In summary, our findings suggest that increased transcription of miRNAs 424 and 503 secondary to thyroid hormone binding to its receptor plays a relevant role in the anti-proliferative and anti-invasive actions of this nuclear receptor in tumor cells. These miRNAs appear to have tumor suppressive actions and could be potential therapeutic targets.

## MATERIALS AND METHODS

### Cells

Human hepatocarcinoma SK-TRb cells and MDA-TRb cells, stably expressing the beta 1 isoform of the thyroid hormone receptor were derived from SK-hep1 and MDA-MB-468 cells, respectively, as previously described [17]. Hep-G2 cells expressing TRb were a kind gift of M. Privalsky. Parental and TRb cells were maintained in Dulbecco's modified Eagle's media (DMEM) supplemented with 10% fetal bovine serum (FBS) depleted of thyroid hormones by treatment with resin AG-1-X8 (Bio-Rad). The human hepatocyte cell line HH4, a gift of J. Campbell and I. Fabregat, was cultured in collagen-coated plates with William's E medium containing 15% fetal calf serum. The cell line THLE-2 derived from primary normal liver cells, a gift of P. Martín, was cultured in Airway Epithelial cell Media (PromoCell ref-21160). When indicated, cells were incubated in serum free medium.

### Retroviral infection

HEK293T cells were cotransfected with 10 µg of pLPCX or pLPCX-TRb that encodes the human TRb isoform, 3.5 µg of VSV, and 6.5 µg of gag-pol constructs (a gift from Dr. P.M. Comoglio) using calcium phosphate. Viral supernatants were harvested 24 and 48 h post-transfection, filtered, and used for infections of HH4 cells in the presence of 4 µg/ml of polybrene. Cells were selected with 2µg/ml puromycin. Pools of resistant cells (HH4-TRb cells) were cultured with thyroid hormone-depleted FBS.

### Plasmids

Fragments +28953/+29193 and +13948/+14644 of the 3'-UTR of the CHEK1 and WEE1 mRNAs with predicted miR-424/503 target binding sequences were amplified by PCR using the primers listed under Supplementary material. These fragments were cloned downstream of the luciferase coding region in pGL3 vector. Reporter plasmids containing the wild-type and mutant 3'-UTRs of Cdc25 and c-Myb were a king gift of A. Dutta and F. Grässer and were described previously [41, 42]. The expression vector for miR-424 (pSGU-miR424) and the corresponding empty vector were a kind gift from I.V. Ramakrishnan [34]. Fragments of the 5'-flanking region of miR424/503 were obtained by PCR and were cloned in pGL3 with the primers listed under Supplementary materials.

### Transfection and reporter assays

Cells, grown in 24 wells plates, were transiently transfected with 300 ng of reporter plasmids and 30 ng of pRL-TK-Renilla (Promega) as a normalizer control, using TransFectin™ (BioRad). When appropriate, the reporter was cotransfected with an expression vector for miR-424 (pSGU-miR424, 200 ng) or with the same amount of an empty vector. Anti-miR Inhibitor for miR-424, miR-503 and the Anti-miR Negative Control#1 were purchased from Ambion (Cat. AM10306 and AM10378). Transfection of miRNA inhibitors was performed using 60 nM of each Anti-miR and TransFectin™ (BioRad) as recommended by the manufacturer. Each experiment was performed in triplicate and was repeated at least 3 times. Data are mean ± S.D and are expressed as fold induction over the values obtained in the control cells.

### Quantitative real time RT-PCR analysis of miRNAs, pri-miRNAs and mRNAs

miR-424 and miR-503 were quantified by the stem-loop real-time PCR, with primers purchased from Applied Biosystems. RNU48 RNA was used as an internal control. Pri-miRNA levels were analyzed by quantitative real-time PCR using specific primers from TaqMan Pri-miRNA Assays (Applied Biosystems). PCRs reactions were detected with FastStartUniversal Sybr Green (Roche). Data analysis was done using the comparative CT method and data were corrected with the RPL19 mRNA levels. Primers used are indicated in the Supplementary material.

### Western blotting

Proteins from cell lysates (20-40 µg) were separated in SDS-PAGE and transferred to PDVF membranes (Immobilon Millipore) that were blocked with 4% BSA. Incubation with primary antibodies was performed overnight at 4°C and with the secondary antibody for 1 h at room temperature. Primary antibodies and the dilution used are listed in the Supplementary material.

### Chromatin immunoprecipitation assays

Cells were plated in 150mm dishes and treated with 5nM T3 for 1h, fixed, lysed following specifications of *17-295 Upstate* kit, and sonicated in a *Bioruptor UCD-200TM* (Diagenode, Belgium). In each immunoprecipitation 4×10^6^ cells and the antibodies listed in the Supplementary material were used. DNA was purified, precipitated and the fragments of miR424/503 promoter amplified with the primers listed under Supplementary material.

### Growth in suspension

Cells were inoculated in the presence or absence of 5nM T3 and kept under agitation under rocking conditions for 24 h. Cells were then plated in 24-wells plates and after 4 h were used for MTT assays. Results were expressed relative to MTT values obtained in cells inoculated in parallel without agitation.

### Flow Cytometry

Floating and adherent cells from triplicate cultures were collected, washed, fixed and centrifuged. Pellets were stained with propidium iodide and sorted in FACScan Q4 (Becton Dickinson, Mountain View, CA) cell sorter. Percentages of cells in sub-G1, G1, S, and G2-M phases were calculated with Mod Fit software for Windows.

### Migration assays

For migration assays, cells were plated in wells containing Culture-Inserts (Ibidi). Twenty-four hours later cells were treated for 2 h with 10µg/ml mitomycin C and inserts were removed. Wound healing was followed for 3 days and photographs were taken at the beginning of the assay (*t*=0 h) and at day 3 at magnification ×100.

### Invasion assays

Cells were inoculated in Transwell plates containing matrigel. Conditioned medium obtained from NIH3T3 cells incubated for 48 h in serum-free-medium and 0.1% bovine serum abumin (BSA) was placed in the outer chamber. Medium containing 0.1% BSA was used as a negative control. Invasion lasted for 16 h, and occurred in the presence and absence of hormone. Filters were fixed with methanol and cells were stained with crystal violet, sealed with Mowiol and scored.

### Xenografts

Groups of athymic nude mice (athymic nude-Nu) 8-10 weeks old were used for xenografting studies. SK-TRb cells (1×10^6^ cells in 100 µl PBS) were injected subcutaneously into each flank, and MDA-TRb cells were orthotopically inoculated into the fat pad of the second abdominal right mammary gland. Cells were implanted in euthyroid mice and in mice made hypothyroid by treatment with 0.02% methymazole and 0.1% potassium perchlorate in the drinking water as described previously [16]. Experiments were carried out following the regulations of the CSIC for animal care and handling (RD 53/2013). Tumors were fixed in 4% buffered formalin and embedded in paraffin wax, and sections (4-5 µm) were stained with Masson's Trichrome. miR-424 and miR-503 levels were analyzed in the tumors 8 weeks after inoculation.

### Extravasation assays in nude mice

Groups of 6 immunodeficient nude mice were injected into the tail vein with 4×10^6^ SK-TRb cells suspended in 100 µl of PBS. Animals were sacrificed and exsanguinated by intra-cardiac puncture at 9 h after injection. Lungs were excised and placed in vials containing 1×PBS. PBS was changed daily for 3 days to eliminate non-incorporated cells. Lungs were divided into 3 pieces (upper, medium and lower) and genomic DNA was extracted from the different segments. Colonization of the organ by the hepatocarcinoma cells was quantitated by real-time PCR of human APO Alu sequences located in human chromosome 11 [51], with the primers listed in the Supplementary material. Results are expressed as the % of amplified human DNA, with respect to the amount of total DNA injected. No amplification of human *Alu* sequences was found in blood, which was used as a negative control.

### Statistical analysis

Data are expressed as means ± S.D. Statistical significance was determined by Student t-test or analysis of variance (ANOVA) followed by the Bonferroni test for experiments with more than two experimental groups. Significance between untreated and T3-treated cells is indicated in the figures as * P<0.05, **P <0.01 and ***P <0.001. All analysis were performed using SPSS (Statistical Software for Social Sciences, Chicago, IL, USA).

## SUPPLEMENTARY FIGURES AND TABLES



## Abbreviations

HAT: histone acetyltransferase
T3: triiodothyronine
TRb: thyroid hormone receptor beta
SK-TRb: SK-hep1 cells stably expressing the TRb isoform
MDA-TRb: MDA-MB-468 cells stably expressing TRb
