# Supplementary material for: microRNAs 424 and 503 are mediators of the anti-proliferative and anti-invasive action of the thyroid hormone receptor beta
Source: Oncotarget. 2014 Apr 2;5(10):2918–33. doi: 10.18632/oncotarget.1577 (PMC4102780; doi:10.18632/oncotarget.1577)
Supplement: Supplementary file 1 [file oncotarget-05-2918-s001.pdf]

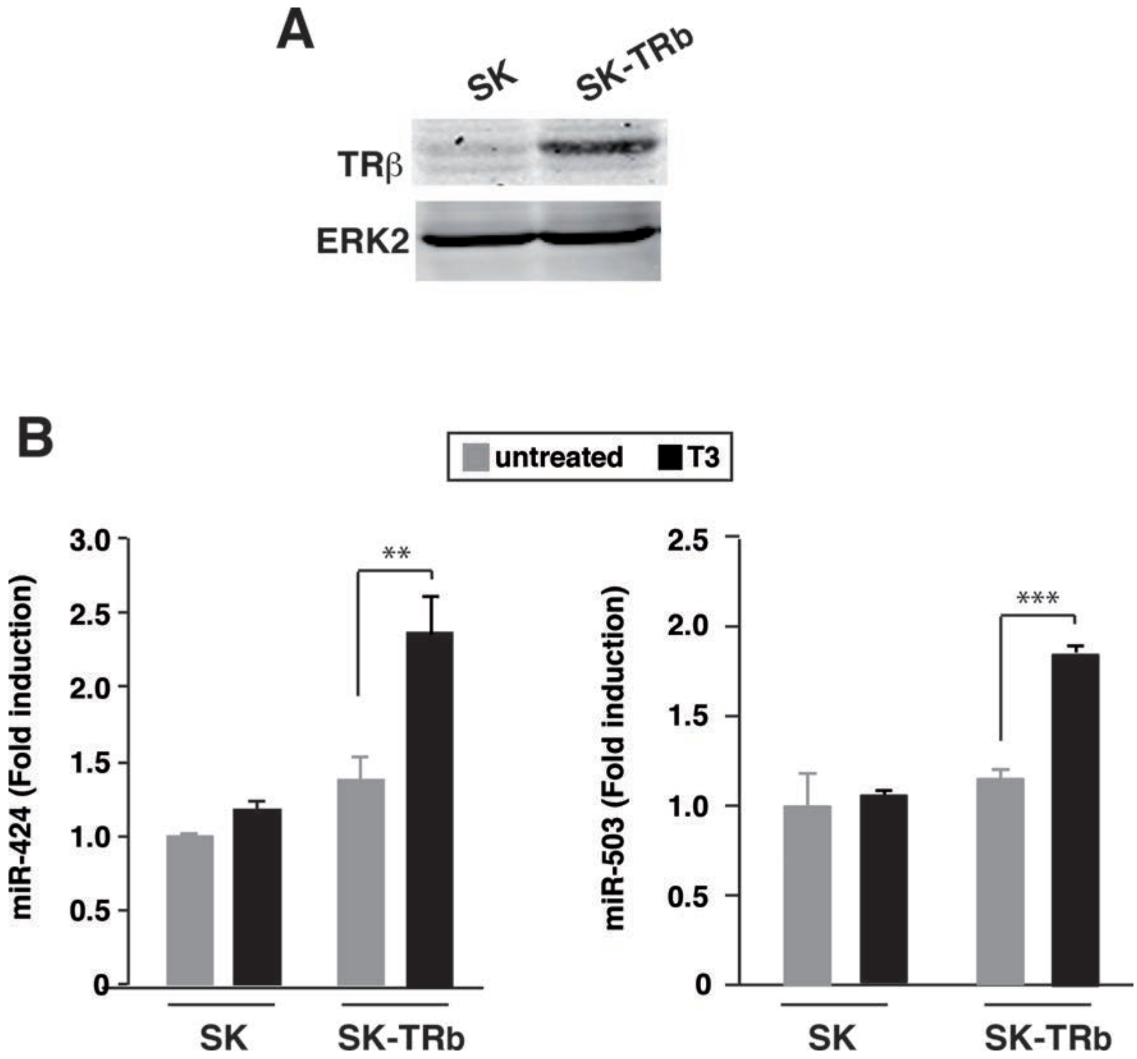

Supplementary Figure S1: (A) The levels of TRb were determined by western blot in parental SK-hep1 hepatocarcinoma cells (SK) and in cells stably transfected with TRb (SK-TRb). ERK2 was used as a loading control. **(B)** miR-424 and miR-503 levels were measured after 36 h of incubation in the presence and absence of 5 nM T3 in SK and SK-TRb cells. Results are expressed as fold induction with respect to the untreated parental cells. Statistically significant differences between untreated and T3-treated cells are indicated with asterisks.

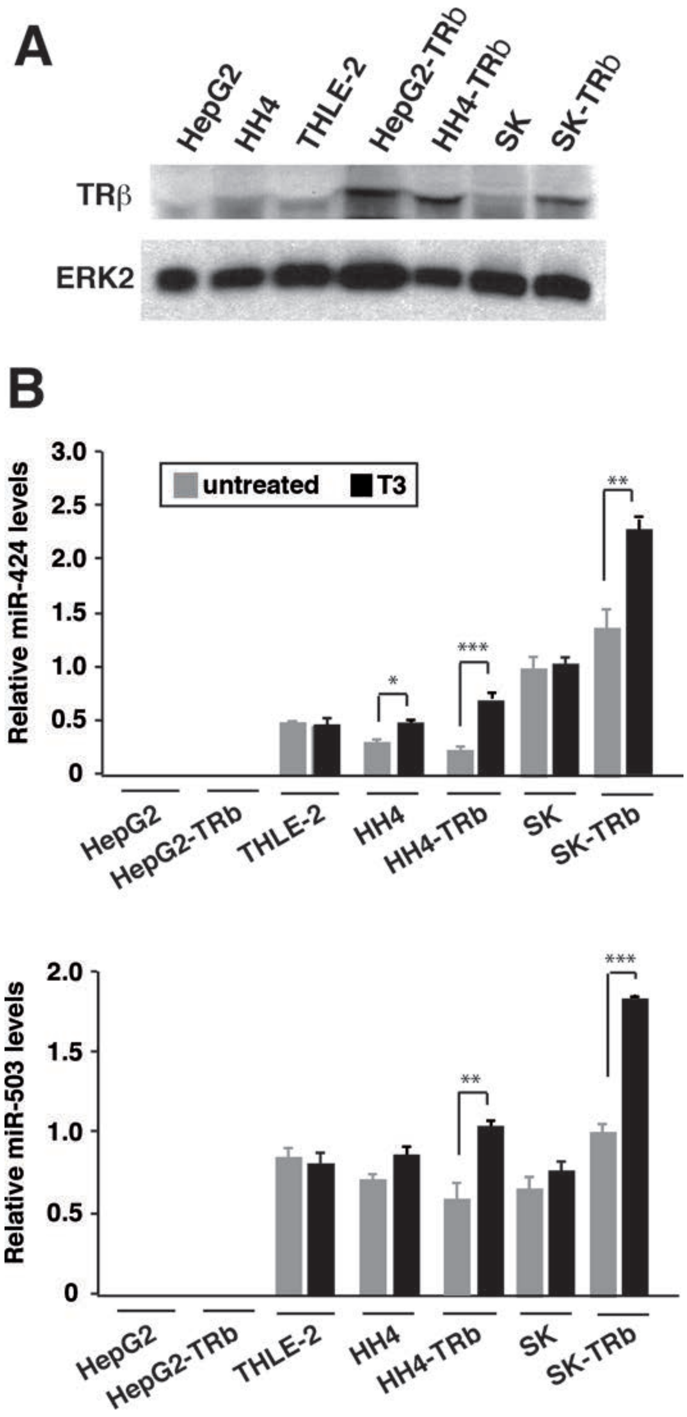

Supplementary Figure S2: (A) The levels of TRb were determined by western blot in the parental cell lines and in cells stably transfected with TRβ ERK2 was used as a loading control. **(B)** miR-424 and miR-503 levels were determined after 36 h of incubation in the presence and absence of 5 nM T3 in the cells indicated. Results are expressed as the percentage to the values obtained in untreated SK-TRb cells. Statistically significant differences between untreated and T3-treated cells are shown with asterisks.

Cdc25 3´-UTR

UGUGGUACUGGGGCUGCUGCUA 660/679
IIIIII
CGACGA seed sequence

GAAGUUACACAGAAAUGCUGCUG 352/370
IIIIII
CGACGA

c-Myb 3´-UTR

AAAACGUUUUUUGCUGCUA 643/661
IIIIII
CGACGA

UUAGCCUGUAGACAUGCUGCUA 667/688
IIIIII
CGACGA

Chk1 3´-UTR

UGGUGAAUAUAGUGCUGCUA 26/45
IIIIII
CGACGA

Wee1 3´-UTR

CCACUGUGGUGGUGUGCUGCUA 251/272
IIIIII
CGACGA

AUUGCCUUGUGAAUUUGCUGCUA 529/551
IIIIII
CGACGA

UAUGUGAAAUGUAUAGCUGCUU 628/649
IIIIII
CGACGA

Supplementary Figure S3: Sequences of the 3´-UTRs of Cdc25, c-Myb, Chk1 and Wee1 mRNAs containing putative binding sites for miR-424 and miR-503. The position of the nucleotides is shown at the right and the seed sequence of the miRNA family annealing with the 3´-UTRs is shown in red.

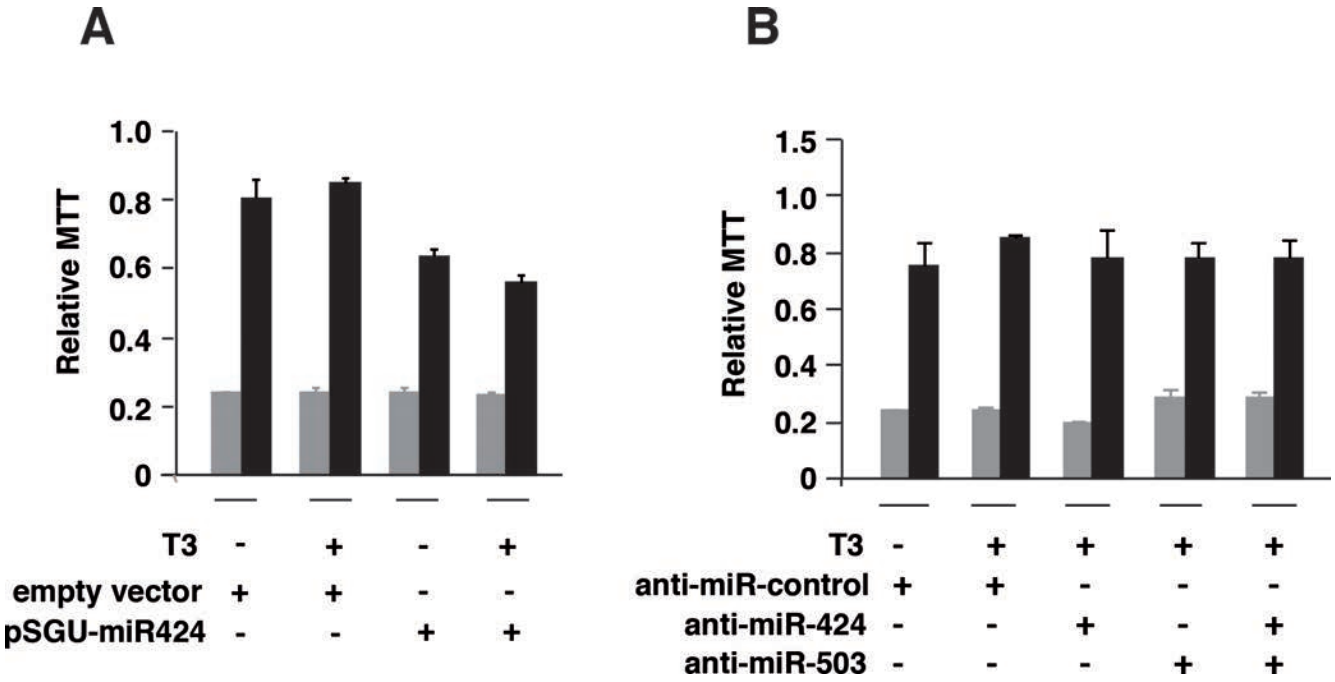

Supplementary Figure S4: (A) HepG2-TRb cells were transfected with the expression vector for miR424 or with an empty vector. After 24h were detached from the plates and grown in suspension under rocking conditions during an additional 24 h period in the presence and absence of T3. Cells were then plated and MTT was assayed. Results were expressed relative to MTT values obtained in cells inoculated in parallel without agitation. (**B**) HepG2-TRb cells were transfected with the indicated antimiRNAs and after 24 h grown in suspension with and without T3 as indicated.

PRIMERS AND ANTIBODIES

PRIMERS

Chk1 3´-UTR (+28953/+29193)
Forward: 5´-CCA GAA GAT TTG GCT TCC TG-3´
Reverse: 5´-CCT TTC CCC AAA GTT TTG CT-3´

Wee1 3´-UTR (+13948/+14644)
Forward: 5´-TGA CAA GAG GAA GCT AGG TTG A-3´
Reverse: 5´-GAA AGG GTG CAT TTT TCC AA-3´

APO
Forward: 5´-AAG TGC TGT AGG CCA TTT AGA TTA G-3´
Reverse: 5´-AGT CTT CGA TGA CAG CGT ATA CAG A-3´

miR424/503 promoter

+59/-382
Forward: 5´-GGC TTC CTT CAG TCA TCC AG-3´
Reverse: 5´-GGA AAC AAT ACC CCA GAG CA-3´

-34/-923
Forward: 5´-AGA GGG CAT GCA TGT GAC TT-3´
Reverse: 5´-GCG GGT GAA TAA AGA CTG GA-3´

-910/-1793
Forward: 5´-GAC AAG ACG CCC CTT TGT AA-3´
Reverse: 5´-ATG CAT GCC CTC TTT TTG TT-3´

-1773/-2574
Forward: 5´-AAC CTT TGC AAA AAG CTT GC-3´
Reverse: 5´-TTA CAA AGG GGC GTC TTG TC-3´

miR424/503 promoter (ChIP assays)

-244/-84
Forward: 5´- TCA CCC ACT ACG TTG TTC CA
Reverse: 5´- GGC AAG GAA TAG GGC AAA AC

-330/-181
Forward: 5´- TTG CCT CTA CTT AAC CTT ATT CGT C
Reverse: 5´- AGC CGA AAG TGT GGC TCT C

-3723/-3911
Forward: 5´-GAA AGA ACG GTA CAA CGA ACG
Reverse: 5´- TTG AAG ATG CAT GCT GAA GTG

ANTIBODIES

Antibody	Dilution	Use	Provider
CDK-6	1:500	WB	Sigma-Aldrich C8343
WEE1	1:1000	WB	Santa Cruz sc-9037
CDC25A	1:500	WB	Santa Cruz sc-7389

(*Continued*)

Antibody	Dilution	Use	Provider
CyclinD2	1:500	WB	Santa Cruz sc-56305
MYB	1:500	WB	Pierce MA5-12339
CHK1	1:1000	WB	Novus Biologicals 19210
E2F3	1:1000	WB	Santa Cruz sc-878
ERK2	1:1000	WB	Santa Cruz sc-154
TRβ	5 μg	ChIP	Santa Cruz sc-737
NCoR	5 μg	ChIP	Santa Cruz sc-8994
CBP	5 μg	ChIP	Santa Cruz sc-369
p/CIP	5 μg	ChIP	Santa Cruz sc-9119
SRC-1	5 μg	ChIP	Santa Cruz sc-6096X
Ac-H4	5 μg	ChIP	Millipore 06-866
